# FHL2 interacts with iASPP and impacts the biological functions of leukemia cells

**DOI:** 10.18632/oncotarget.16617

**Published:** 2017-03-28

**Authors:** Wenting Lu, Tengteng Yu, Shuang Liu, Saisai Li, Shouyun Li, Jia Liu, Yingxi Xu, Haiyan Xing, Zheng Tian, Kejing Tang, Qing Rao, Jianxiang Wang, Min Wang

**Affiliations:** ^1^ State Key Laboratory of Experimental Hematology, Institute of Hematology and Blood Diseases Hospital, Chinese Academy of Medical Sciences & Peking Union Medical College, Tianjin 300020, China

**Keywords:** FHL2, iASPP, biological functions, leukemia

## Abstract

iASPP is an inhibitory member of apoptosis-stimulating proteins of p53 (ASPP) family, which inhibits p53-dependent apoptosis. iASPP was highly expressed in acute leukemia, inhibited leukemia cells apoptosis and promoted leukemogenesis. In order to clarify its mechanism, a yeast two-hybrid screen was performed and FHL2 was identified for the first time as one of the binding proteins of iASPP. FHL2 was highly expressed in K562 and Kasumi-1 cells. FHL2 and iASPP interacted with each other and co-localized in both nucleus and cytoplasm. Either FHL2 or iASPP silenced could reduce cell proliferation, induce cell cycle arrest at G0/G1 phase, and increase cell apoptosis. Western blot analysis showed that the level of p21 and p27 increased, CDK4, E2F1, Cyclin E and anti-apoptotic proteins Bcl-2 and Bcl-xL reduced. Interestingly, when FHL2 was knocked down, the protein expression level of iASPP also decreased. Similarly, the expression of FHL2 would reduce when iASPP was silenced. These results indicated that FHL2 might be a novel potential target for acute myelocytic leukemia treatment.

## INTRODUCTION

Leukemia is usually caused by genetic translocations, gene overexpression and gene mutations. It is known that p53 is one of the most common mutant genes in human cancer, however, only 10-15% of p53 mutations were found in leukemia [1]. Thus, it was supposed that there might be an abnormal gene regulation of p53. ASPP1, ASPP2 and iASPP belong to apoptosis-stimulating proteins of p53 (ASPP) family [2]. The three members of ASPP family can interact with p53 and modulate its behavior. ASPP1 and ASPP2 enhance the ability of p53 to induce apoptosis [3, 4], iASPP is an inhibitory member of ASPP, which inhibits p53-dependent apoptosis [5]. Our previous study revealed that iASPP was highly expressed in patients with acute leukemia (AL) [6]. In addition, iASPP was considered as an oncogene in enhancement of self-renewal and resistance to irradiation and chemotherapy of hematopoietic stem cells [7]. In order to clarify the mechanism of iASPP in leukemogenesis and looking for its cooperating partner, a yeast two-hybrid screen was performed, and FHL2 was identified as a novel binding partner of iASPP.

Four and a half LIM domains 2 (FHL2), also known as DRAL or SLIM3, belongs to FHL2 family [8]. FHL2 can function as adaptors to mediate protein-protein interactions because of the LIM domain. Besides, FHL2 shuttles between cytosol and nucleus, and plays important roles in the regulation of signal transduction [9–12], cell survival [13, 14], motility [15, 16] and adhesion [17, 18]. Interestingly, FHL2 is upregulated in many cancers compared with normal tissues, for example, human melanoma, breast cancer and pancreatic cancer [11, 19, 20]. It is notable that FHL2 also highly expressed in acute erythroid leukemia (AML-M6) [21], indicating that FHL2 may play a vital role in leukemogenesis, especially in the type of AML-M6. Since iASPP communicates with FHL2, what are their functions in AML?

In order to confirm the relationship between iASPP and FHL2, the expression level of FHL2 and iASPP in several types of AML cells was examined, and their interaction domains were further investigated. The results indicated that both iASPP and FHL2 highly expressed in some leukemia cell lines, when silenced either FHL2 or iASPP could reduce the cell proliferation, induce cell cycle arrest at G0/G1 phase, and increase cell apoptosis via p21 and Bcl-2 signaling pathways. In addition, there was a correlation between the expression level of FHL2 and iASPP. It is the first report to show a novel role of the interaction between iASPP and FHL2 in leukemogenesis.

## RESULTS

### L1/2-3 domain of FHL2 is required for the interaction with iASPP

**Figure 1:**
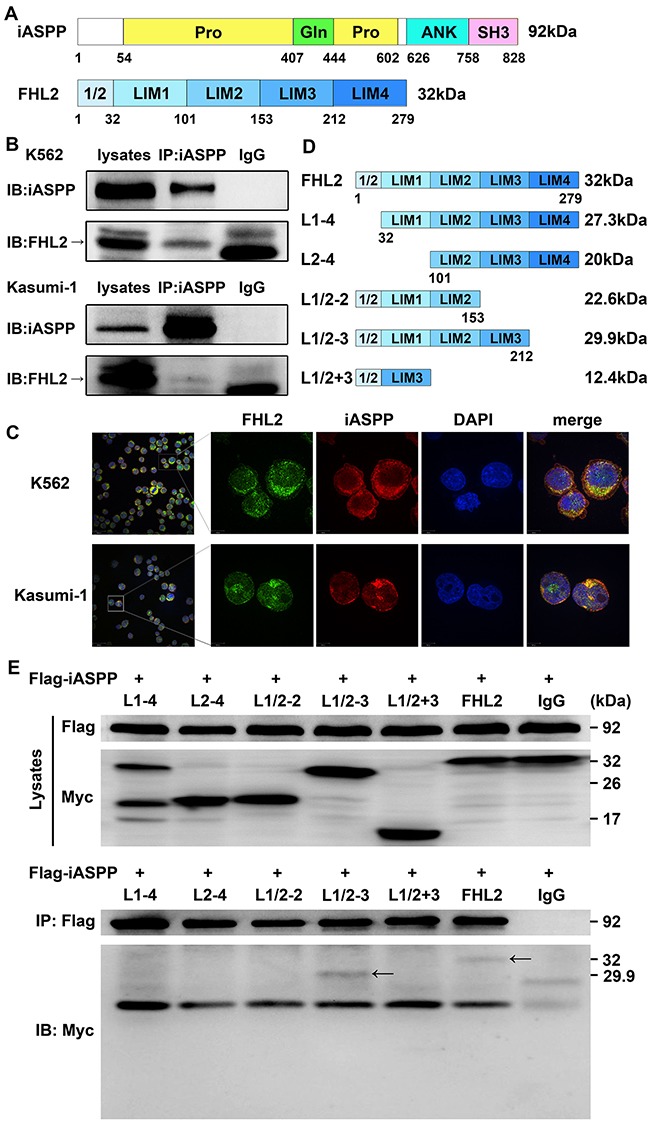
Identification of FHL2 as a novel binding partner of iASPP **(A)** The schematic diagram of iASPP and FHL2. **(B)** K562 and Kasumi-1 cells were harvested and lysed with lysis buffer. Cell lysates were immunoprecipitated with anti-iASPP or anti-IgG antibodies. Immunoblot analyses were performed with indicated antibodies. **(C)** Co-localization analyses of FHL2 and iASPP were performed in K562 and Kasumi-1 cells by immunofluorescence. Anti-FHL2 and anti-iASPP antibodies were used as primary antibodies, and DAPI was used for nuclear staining. Bars represent 8 μm. **(D)** Different truncation mutants of FHL2. **(E)** Flag-iASPP and Myc-FHL2 or different truncation mutants of FHL2 expression plasmids were co-transfected into HEK293T cells. 48 hours after transfection, cell lysates were prepared and immunoprecipitated with anti-Flag or anti-IgG antibodies. Immunoblot analyses were performed using anti-Flag or anti-Myc antibodies. Arrows indicate Myc-L1/2-3 and Myc-FHL2 could coimmunoprecipitate with Flag-iASPP. Lysates, nonimmunoprecipitated cell lysates; IP, immunoprecipitation; IB, immunoblotting; IgG, control IP with isotype antibody.

Our previous yeast two-hybrid analysis using iASPP as bait indicated that FHL2 might be a binding partner of iASPP. The protein of iASPP is comprised of proline-rich (Pro), glutamine-rich (Gln), ankyrin repeats (ANK) and Src homology 3 (SH3) domains, and the FHL2 protein consists of four and a half LIM domains (Figure 1A). To confirm the interaction between FHL2 and iASPP, co-immunoprecipitation (Co-IP) and immunoblotting analysis were performed. The results showed that FHL2 could co-immunoprecipitate with iASPP in K562 and Kasumi-1 cells (Figure 1B). Immunofluorescence analysis further confirmed that FHL2 and iASPP interacted with each other and co-localized in both nucleus and cytoplasm in above two cell lines (Figure 1C). In addition, the binding domain of FHL2 and iASPP interaction was further investigated. The Flag-tagged iASPP and different truncation mutants of Myc-tagged FHL2 were co-transfected into HEK293T cells (Figure 1D). After 48 hours culture, Co-IP assays were performed, and the results showed that the full length and truncation mutant contained the first 3 and a half LIM domains (L1/2-3) could interact with iASPP (Figure 1E).

### FHL2 is highly expressed in some leukemia cell lines

**Figure 2:**
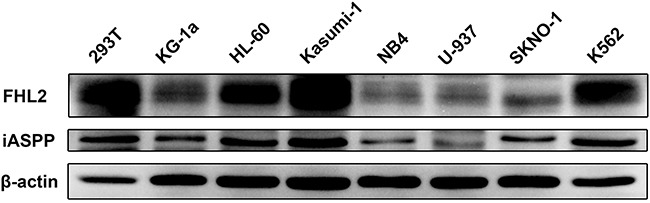
FHL2 expression levels in AML cell lines Expression level of FHL2 and iASPP was assessed by Western blot in AML cell lines using the indicated antibodies. β-actin was used as an internal control.

As confirmed above that FHL2 could interact with iASPP, whether the interaction between these two proteins would play a role in leukemogenesis was then investigated. The expression levels of these two proteins in leukemia cells and the correlation between their expression levels were examined. The expression level of FHL2 in AML cell lines was analyzed, and the result revealed a high expression level of FHL2 in K562, Kasumi-1 and HL-60 cell lines by Western blot assay (Figure 2). Interestingly, the expression level of iASPP is highly consistent with that of FHL2.

### FHL2 silencing reduces cell proliferation, induces cell cycle arrest at G0/G1 phase and increases cell apoptosis

**Figure 3:**
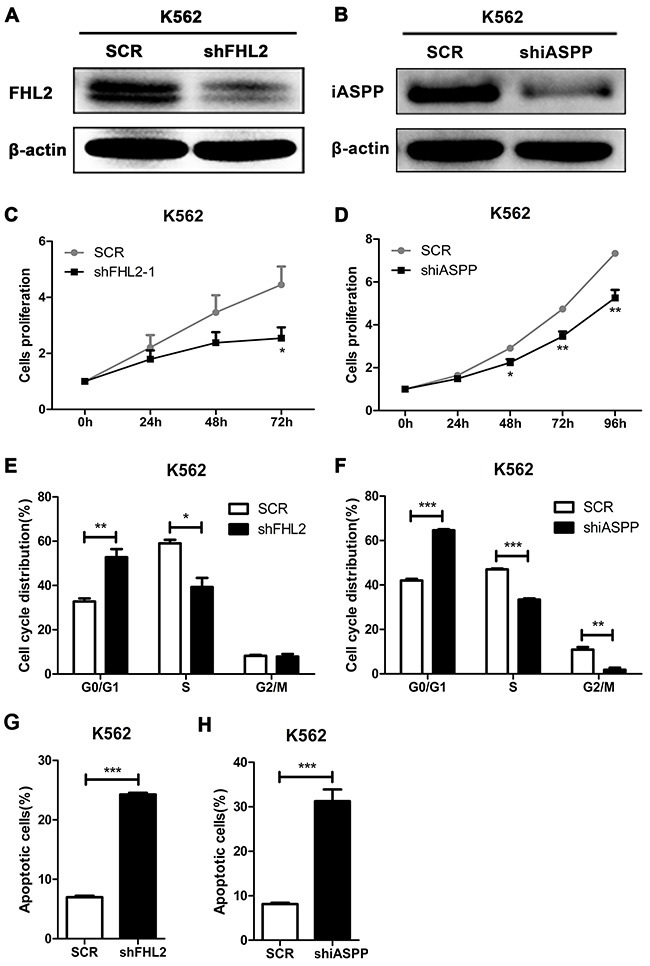
Silence of FHL2 or iASPP in K562 cells reduces cell proliferation, induces cell cycle arrest at G0/G1 phase, and increases cell apoptosis **(A, B)** After infected with lentivirus of shFHL2 or shiASPP for 72 hours in K562 cells, the expression level of FHL2 or iASPP was measured by Western blot. β-actin was used as an internal control. **(C, D)** MTT assays were used to assess cell proliferation ability after 24 hours of lentivivus infection in K562 cells. **(E, F)** Cell cycles of K562 cells with FHL2 or iASPP knockdown were analyzed by flow cytometry after PI staining. **(G, H)** Cell apoptosis was analyzed in K562 cells after FHL2 or iASPP knockdown by flow cytometry using Annexin-V/PI staining. Data represents there independent experiments (*P<0.05, **P<0.01, ***P<0.001). SCR, cramble sequence.

**Figure 4:**
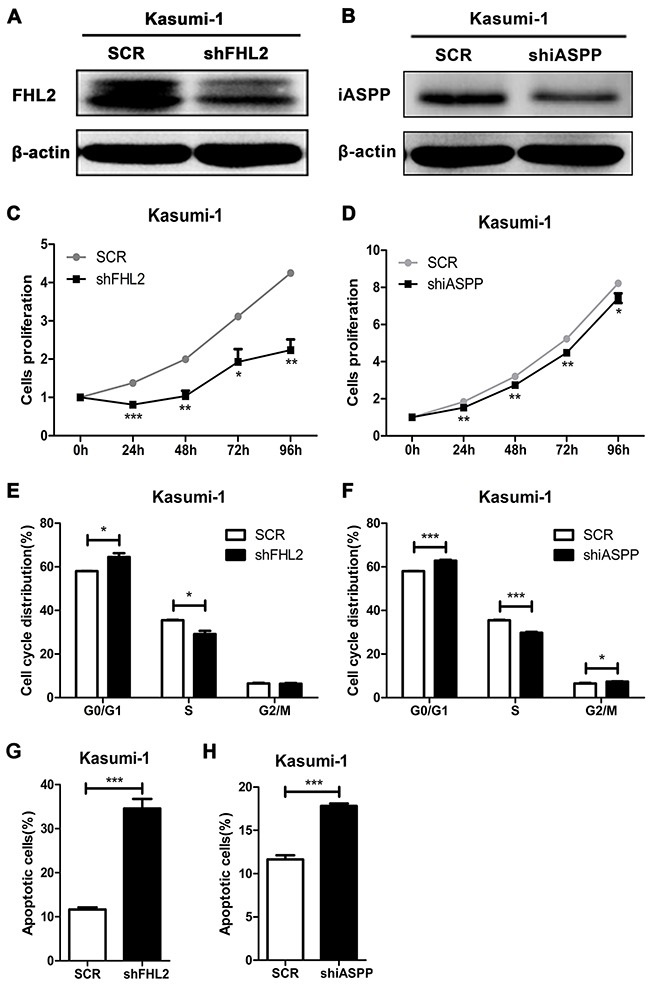
FHL2 or iASPP knockdown in Kasumi-1 cells inhibits cell growth, increases the percentage of cells in G0/G1 phase and cell apoptosis **(A, B)** Western blot was performed to examine the expression level of FHL2 or iASPP in Kasumi-1 cells after FHL2 or iASPP silencing for 96 hours. **(C, D)** Cell growth of Kasumi-1 cells with FHL2 or iASPP knockdown was measured by MTT assays. **(E, F)** Flow cytometry was used to analyze cell cycle of Kasumi-1 cells when FHL2 or iASPP was knocked down. **(G, H)** After Annexin-V/PI staining, cell apoptosis was analyzed by flow cytometry in Kasumi-1 cells with FHL2 or iASPP knockdown. Data represent there independent experiments (*P<0.05, **P<0.01, ***P<0.001).

As stated above that FHL2 highly expressed in some leukemia cell lines, FHL2 knockdown experiment was performed to address its role in leukemogenesis. The most efficient silencing sequence of FHL2 was screened and used for following experiments. Lentiviral constructs encoding scramble sequence (SCR) or target sequence of FHL2 (shFHL2) was transfected into K562 and Kasumi-1 cells. Western blot analysis was used to confirm the silencing efficiency. An obvious reduction in FHL2 protein expression was observed in shFHL2 transfected K562 and Kasumi-1 cells compared to SCR transfected cells, respectively (Figure 3A, 4A). To detect the effect of FHL2 silencing on the proliferation of K562 and Kasumi-1 cells, MTT assays were performed. As shown, the proliferation of shFHL2 transfected cells was significantly slower than that of SCR transfected cells (Figure 3C, 4C). Then flow cytometry assay was performed to identify whether the cell cycle and cell apoptosis were involved in the decreased growth in shFHL2 transfected cells. A significant increase in the proportion of cells in G0/G1 phase, decrease in S phase and increase in apoptosis were observed in K562 and Kasumi-1 cells respectively after FHL2 knockdown (Figure 3E, 4E, 3G, 4G). These results suggested that FHL2 might work as an oncogene in AML.

### iASPP knockdown has similar effects on AML cells as that of FHL2 knockdown

We had confirmed that iASPP interacted with FHL2, and knockdown of FHL2 expression could play a role on cell proliferation, cell cycle and cell apoptosis in leukemia cells. To address what would happen if iASPP was silenced, knockdown experiment was performed, and Figure 3B and 4B showed the efficiency of iASPP silencing in K562 and Kasumi-1 cells respectively by Western blot analysis. MTT assay demonstrated that the growth of cells was slower in shiASPP transfected cells than that of SCR transfected cells (Figure 3D, 4D). Flow cytometry assay showed that iASPP silencing induced cell cycle arrest in G0/G1 phase and reduced the cell proportion in S phase (Figure 3F, 4F). Moreover, the apoptosis ratio of shiASPP transfected cells was higher than that of SCR transfected cells (Figure 3H, 4H). Above results indicated that silence of either FHL2 or iASPP expression had a similar effect on leukemia cells, which suggested that both of them might play an important role in leukemogenesis.

### The expression correlation was found between FHL2 and iASPP

**Figure 5:**
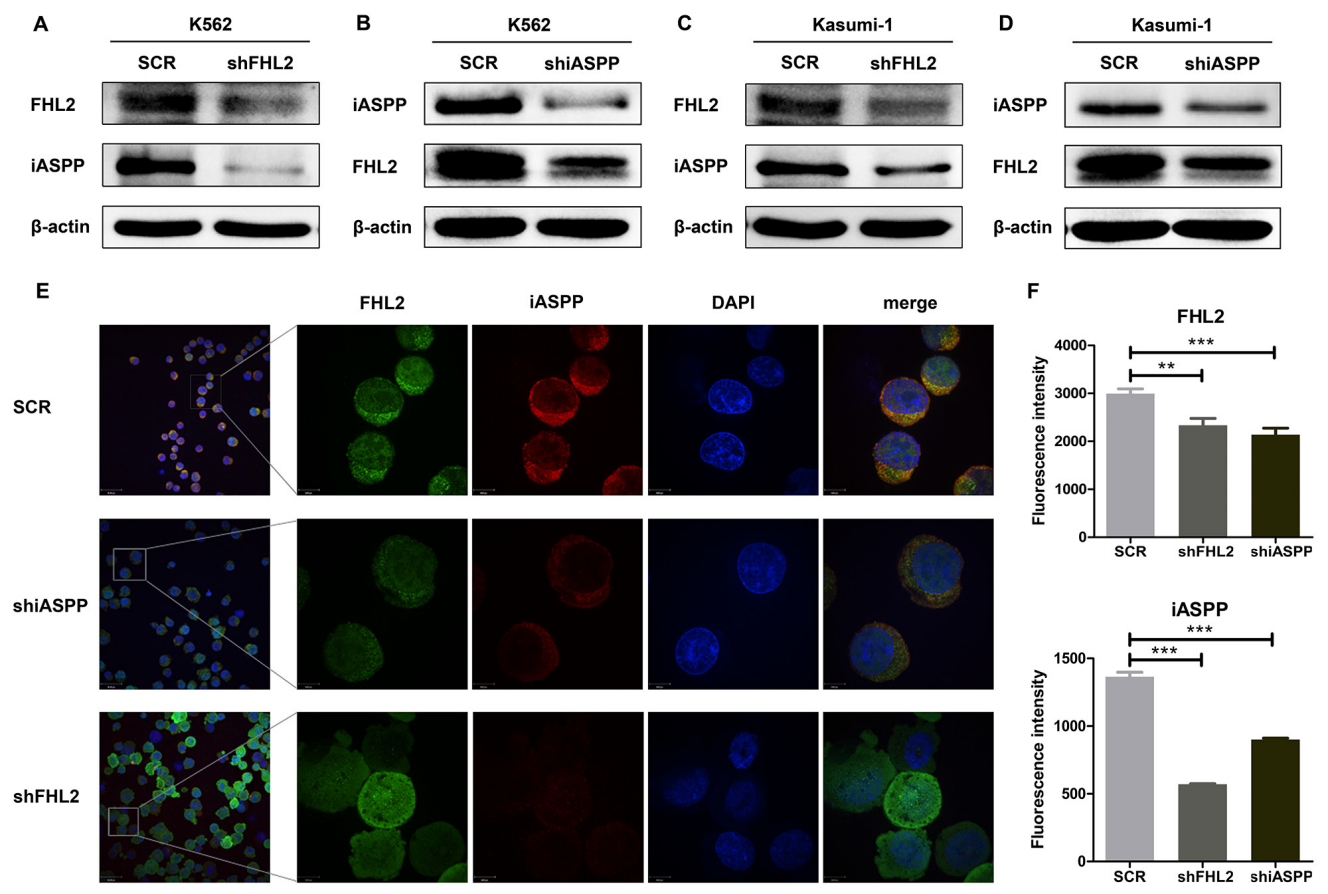
The correlation between the expression level of FHL2 and iASPP **(A, C)** The expression level of iASPP was detected when FHL2 was knocked down in K562 and Kasumi-1 cells; **(B, D)** the expression level of FHL2 was assessed after iASPP knockdown in K562 and Kasumi-1 cells. Western blots were performed using indicated antibodies. **(E)** Immunofluorescence analysis was performed to detect the fluorescence intensity of FHL2 and iASPP after iASPP or FHL2 knockdown in K562 cells. Anti-FHL2 and anti-iASPP antibodies were used as primary antibodies, and DAPI was used for nuclear staining. Bars represent 8 μm. **(F)** Representative histograms of fluorescence intensity are displayed, data represent ten positive cells (**P<0.01, ***P<0.001).

As we stated before that iASPP interacted with FHL2, and knockdown either of the protein expressions possessed the similar effects on AML cells, which promoted us to explore whether silence one of the interacting proteins would interfere the expression or the stability of the other protein. Interestingly, it was found that when knocked down FHL2 expression, the expression level of iASPP also decreased in both K562 and Kasumi-1 cells (Figure 5A, 5C). Similarly, the expression level of FHL2 would reduce after iASPP was silenced (Figure 5B, 5D). This phenomenon was further demonstrated by immunofluorescence analysis in K562 cells (Figure 5E). It showed that the expression level of one of the proteins was knocked down, the expression of the other one would decrease, too. The fluorescence intensity of FHL2 or iASPP was analyzed by ImageJ software (Figure 5F).

### Both FHL2 and iASPP silencing affect p21 and Bcl-2 signaling pathways

**Figure 6:**
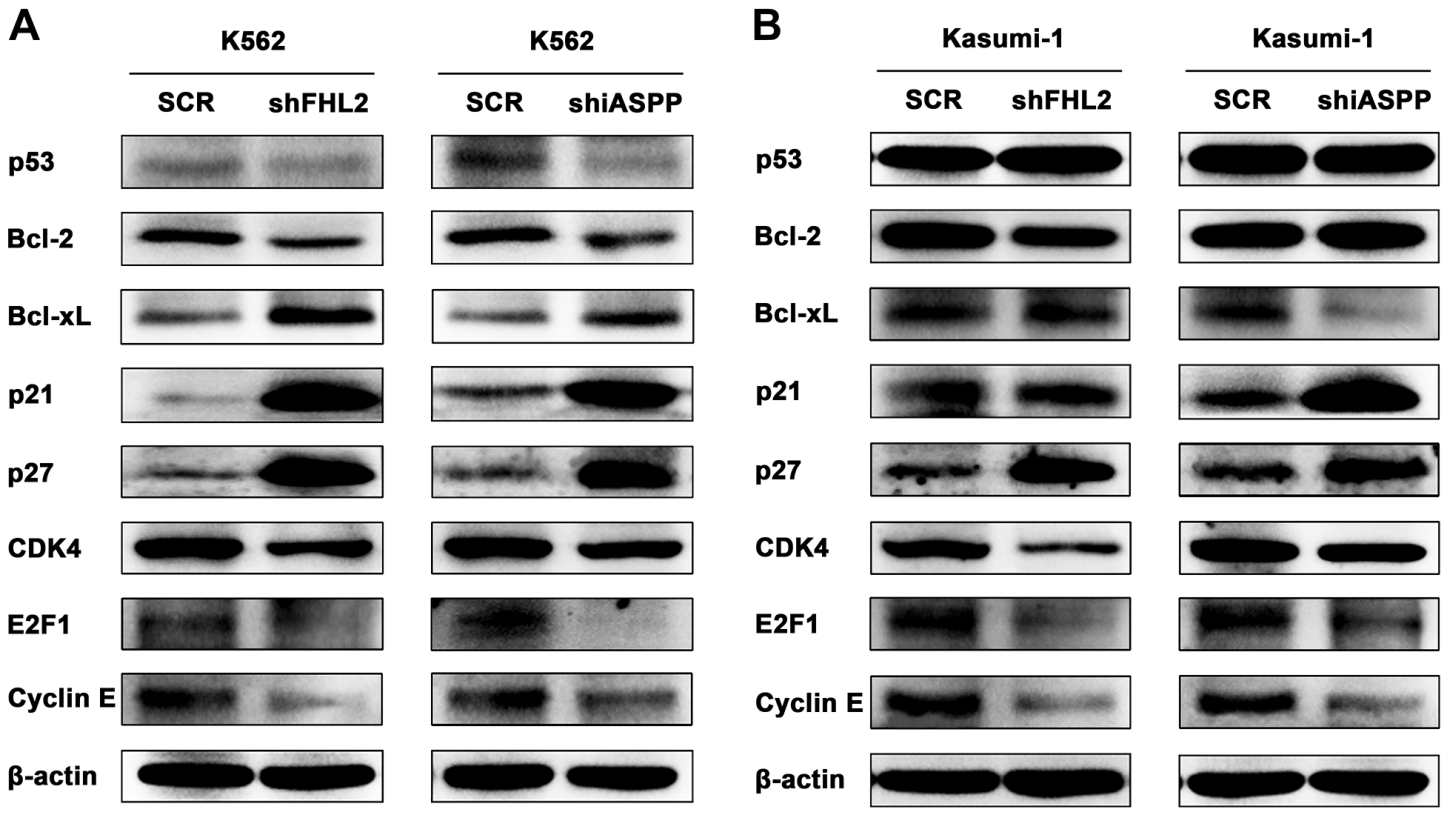
Knockdown of either FHL2 or iASPP could affect the p21 and Bcl-2 signaling pathways in AML cells The expression level of p53, Bcl-2, Bcl-xL, p21, p27, CDK4, E2F1 and Cyclin E were detected by Western blot using indicated antibodies in K562 **(A)** and Kasumi-1 **(B)** cells after either FHL2 or iASPP silencing. β-actin was used as an internal control.

Finally the signal pathways that might be involved in the cell proliferation, cell cycle and cell apoptosis related to FHL2 and iASPP knockdown were investigated. The expression level of cell cycle regulators and anti-apoptotic proteins were analyzed by Western blot assay. It showed that the expression level of p21 and p27 increased significantly, CDK4, E2F1 and Cyclin E decreased in both shFHL2-transfected and shiASPP-transfected cells (Figure 6). At the same time, the expressing level of anti-apoptosis proteins Bcl-2 and Bcl-xL were reduced. However, in Kasumi-1 cells, there was no obvious change in the expression level of p21, Bcl-xL or Bcl-2 when FHL2 or iASPP silenced, respectively (Figure 6B), which may be due to different cell contexts between K562 and Kasumi-1 cells. It was supposed that other signaling pathways without p53 might be activated, because the protein level of p53 had no obvious change. These data indicated that the p21 and Bcl-2 signaling pathways were involved in the regulatory mechanisms associated with FHL2 and iASPP.

## DISCUSSION

In recent years, iASPP has been increasingly studied in many cancers, including human hepatocellular carcinoma [22], human glioblastoma [23], gastric cancer [24], and so on. Our previous study showed that iASPP was highly expressed in leukemia patients than that of normal controls [6], down-regulation of iASPP expression in leukemia cells by RNA interference could induce p53-dependent apoptosis of leukemia cells [25], over-expression of iASPP in transgenic mice model could increase the population, self-renewal capacity, resistance to irradiation and chemotherapy of hematopoietic stem cells [7]. However, there are still many issues to be explored in iASPP study, such as how iASPP is regulated by other proteins, how different iASPP splicing isoforms coordinate to play a role in cancer cells. Therefore, the binding proteins of iASPP were screened by yeast two-hybrid technique, and FHL2 was found to be one of the iASPP binding proteins for the first time. In this study, we confirmed that the truncation mutant contained the first 3 and a half LIM domains (L1/2-3) of FHL2 was required for the interaction with iASPP through Co-IP assays. Immunofluorescence analysis further demonstrated the interaction between FHL2 and iASPP, and both of them localized in nucleus and cytoplasm in leukemia cells.

FHL2 has been mainly studied in solid tumors in past years. The expression level and function of FHL2 are significantly different in various types of cancers, indicating that FHL2 works either as an oncogene or as a tumor suppressor gene in the occurrence and development of cancers [19, 26–30], depending on different communicating partners. As stated above, high expression of iASPP in leukemia cells could inhibit cell apoptosis and promote cell proliferation, then we wanted to address whether this effect was dependent on the interaction of FHL2, and down-regulated the expression of iASPP would affect that of the other and vise verse? It was found that knockdown either FHL2 or iASPP in K562 and Kasumi-1 cells could lead to similar effects: cell proliferation reduced, cell cycle arrested at G0/G1 phase and cell apoptosis increased. And an interesting phenomenon was observed in both cells that when knocked down FHL2 expression, the expression level of iASPP also decreased and if iASPP was silenced, the expression level of FHL2 decreased, too. Immunofluorescence analysis in K562 cells further demonstrated this phenomenon. Because FHL2 always functions as an adaptor to mediate protein-protein interactions and induces some biological functions [31, 32], we propose that FHL2 possibly acts as an adaptor to mediate the degradation of iASPP. It is likely that other protein may participate in the degradation, which needs to be further studied.

According to the biological changes of leukemia cells after FHL2 or iASPP knockdown, the expression level of cell cycle regulators and Bcl-2 family members were analyzed by Western blot assay. The expression level of p21 and p27 significantly increased and that of CDK4, E2F1, Cyclin E, Bcl-2 and Bcl-xL reduced. These results indicated that p21 and Bcl-2 signaling pathways were involved in the regulatory mechanisms associated with FHL2 and iASPP. However, there is no obvious change in P53 expression level, which may be due to that p53 is mutant in K562 and Kasumi-1 cells. Li found that G0/G1 cell cycle arrest and p21^Waf1/Cip1^ up-regulation when iASPP was silenced in p53-mutant glioblastoma cell line U251 [23]. Dual luciferase assays were also conducted to investigate the effect of iASPP on p53 transactivation (data not shown). Results showed that P53 could increase the transcriptional activity of p21^Waf1/Cip1^, which did not show any obvious change when iASPP was added. Sebastien Gillotin also got the similar results [33]. It is notable that in dual luciferase assays a wild type of p53 was used, but in K562 and Kasumi-1 cells p53 was mutant. The function of wild-type or mutant p53 is likely to be different. All above suggests there may be other signaling pathways involved in p21^Waf1/Cip1^ transactivation regulated by iASPP when p53 is mutant.

In conclusion, the interaction between iASPP and FHL2 was reported for the first time. Cell proliferation decreasing, cell cycle arresting at G0/G1 phase, and cell apoptosis increasing occurred via p21 and Bcl-2 signaling pathways when either FHL2 or iASPP was silenced. The results may help to understand the role of FHL2 and iASPP interaction in leukemia cells, and provide a novel therapeutic target for AML.

## MATERIALS AND METHODS

### Immunoprecipitation (Co-IP) and immunoblotting analysis

K562, Kasumi-1 cells (2×10^7^) and HEK293T cells (3×10^7^) transient expressing full-length of iASPP and FHL2 or different truncation mutants of FHL2 were harvested and lysed in 1ml of lysis buffer (20mM Tris PH7.5, 150mM NaCl, 1% Triton X-100, 2.5mM sodium pyrophosphate, 1mM EDTA, 1% Na_3_VO_4_, 0.5ug/ml leupeptin, 1mM phenylmethanesulfonyl fluoride). The lysate was cleared by centrifugation, followed by incubation with anti-iASPP monoclonal antibody (Abcam, UK) or the control anti-IgG antibody (Beyotime Biotechnology, China) overnight at 4°C, and then incubated with protein A/G-Sepharose beads (Santa Cruz Biotechnology, USA) for 4h at 4°C. The beads were washed five times with lysis buffer. Proteins were separated and transferred onto polyvinylidene difluoride membranes, blotted with anti-iASPP (Abcam, UK), anti-FHL2 (Abcam, UK; Marine Biological Laboratory, USA), or anti-Flag (Sigma, Germany) and anti-Myc (Cell Signaling Technology, USA) antibodies by using SuperSignal chemiluminescence detection system (Pierce, USA). Furthermore, proteins collected from cells with iASPP or FHL2 silencing were immunoblotted with the following primary antibodies: anti-p53 (Santa Cruz Biotechnology, USA), anti-p21 (Cell Signaling Technology, USA), anti-p27 (Cell Signaling Technology, USA), anti-Bcl-2 (Cell Signaling Technology, USA), anti-Bcl-xL (Cell Signaling Technology, USA), anti-CDK4 (Cell Signaling Technology, USA), anti-E2F1 (Cell Signaling Technology, USA) and anti-Cyclin E (Cell Signaling Technology, USA). Anti-β-actin (Sigma, Germany) was used as an internal control.

### Cell culture

HEK293T cells were cultured in Dulbecco's modified Eagle medium (DEME) (Sigma, Germany) supplemented with 10% fetal bovine serum (HyClone, USA). K562 cells were cultured in RPMI 1640 (Sigma, Germany) with the same supplements; Kasumi-1 cells were cultured in RPMI-1640 with 20% FBS (Gibco, USA). Cells were maintained in a 5% CO_2_ humidified incubator at 37°C.

### Plasmid constructs and transduction

The human full-length cDNA of iASPP was cloned from pcDNA3.1-WT-iASPP-V5 (a kind gift from Prof. Lu Xin, Ludwig Institute for Cancer Research, Oxford, UK) into pcDNA3.1-Flag expression vector (Invitrogen, USA). Full-length cDNA and different truncation mutants of FHL2 were cloned from cDNA of HEK293T cells into pcDNA3.1-Myc expression vector (Invitrogen, USA). Plasmids were co-transfected into HEK293T cells using polyethylenimine (PEI) (Polyscience, USA). Specific primers for iASPP and FHL2 were synthesized by Invitrogen (Table 1). FHL2 specific (shFHL2) and scrambled control (SCR) shRNAs were designed by Ambion software. The target sequence of FHL2 was 5’-CGACTGCTTTAACTGTAAGAA-3’ and the scramble sequence was 5’-CAACAAGATGAAGAGCACCAA-3’. Fragments were inserted into the PLKO.1-GFP vector. Lentiviral vector constructs and packaging vectors (pCMV and pMDG) were co-transfected into HEK293T cells using PEI to produce lentivirus.

**Table 1:** The details of primers of target genes for PCR

Gene	Primers (5’-3’)	Product (bp)
iASPP	F: CCGGAATTCCATGGACAGCGAGGCATTCC	2506
	R: CCGCTCGAGCTAGACTTTACTCCTTTGAGGC	
FHL2	F: CCGGAATTCATGACTGAGCGCTTTGACTG	840
	R: CCCAAGCTTGATGTCTTTCCCACAGTCG	
L1-4	F: CCGGAATTCATGACCCTGTTCGCCAACACC	738
	R: CCCAAGCTTGATGTCTTTCCCACAGTCG	
L2-4	F: CCGGAATTCATGAAGTGCCAGGAATGCAAG	540
	R: CCCAAGCTTGATGTCTTTCCCACAGTCG	
L1/2-2	F: CCGGAATTCATGACTGAGCGCTTTGACTG	501
	R: CGCGGATCCCTGCATGGCATGTTGTTT	
L1/2-3	F: CCGGAATTCATGACTGAGCGCTTTGACTG	678
	R: CGCGGATCCCTTCTTGGCATACAAGTC	
L1/2+3	F: CCGGAATTCATGACTGAGCGCTTTGACTG	276
	R: CGCGGATCCCTTCTTGGCATACAAGTC	
	Overlap-F: CCAACGAGTACTCATCCTGCGTTCAGTGCAAAAAGC	
	Overlap-R: GCTTTTTGCACTGAACGCAGGATGAGTACTCGTTGG	

### MTT assay

Cell proliferation was measured by MTT assays after 24 hours of lentivirus infection. Cells were seeded in 100μl RPMI 1640 medium containing 10% FBS into 96-well plates at 1×10^4^/well for 0, 24, 48, 72 or 96 h. 10μl of 5 mg/ml MTT (Sigma, Germany) was added to the wells for each time point, and the plates were incubated at 37°C for 4 hours. Then 100μl of 0.1N HCl in 10%SDS was added to stop the reaction. The absorbance value at 546 nm was measured by Synergy H4 Hybrid Microplate Reader (BioTek, USA) after an overnight incubation.

### Cell cycle analysis

1×10^6^ GFP^+^ cells were collected and fixed in 1ml 75% ethanol at 4°C for 24 hours and then incubated with 10 μg/ml RNase A for 10 minutes at room temperature. After that, cells were stained with 50 μg/ml propidium iodide (PI) at room temperature for 10 minutes. The cell cycle distribution was assayed with FACS LSRII flow cytometer (BD Bioscience, USA) and analyzed using ModFit software.

### Apoptosis measurement by AnnexinV/PI staining

5×10^5^ GFP positive cells were washed with 1×AnnexinV binding buffer twice and resuspended in 100μl binding buffer, and then added AnnexinV-Alexa 647 and PI (Biolegend, USA) for 15 minutes at room temperature. All samples were measured by FACS LSRII.

### Immunofluorescence analysis

The target cells were fixed with 4% paraformaldehyde for 15 minutes, permeabilized by 0.25% TritonX-100 for 10 minutes and sequentially incubated with indicated primary antibodies, secondary antibodies (DyLight 649 goat anti-mouse IgG antibody, DyLight 488 donkey anti-rabbit IgG antibody) (Biolegend, USA), and 4-6-diamidino-2- phenylindole (DAPI) (Invitrogen, USA). Images were detected by the confocal imaging system (PerkinElmer, USA). The fluorescence intensity was analyzed by ImageJ software.

### Statistical analysis

Data obtained from triplicated experiments were expressed as the mean ± SD and analyzed using SPSS software. The significance of differences between two groups was determined using an independent-sample *T* test. A *p* value less than 0.05 was considered statistically significant; asterisks indicate significant differences (*P < 0.05; *P < 0.01; *P < 0001).

## Abbreviations

FHL2: four and a half LIM domains 2
ASPP: apoptosis-stimulating proteins of p53
iASPP: inhibitory member of ASPP family
AL: acute leukemia
AML: acute myeloid leukemia
AML-M6: acute erythroid leukemia
BMMNCs: bone marrow mononuclear cells
Co-IP: coimmunoprecipitation
L1/2-3: the first 3 and a half LIM domains
SCR: scramble sequence
shFHL2: target sequence of FHL2
